# A case-control study of end-of-life antimicrobial use in Non-hospitalized hospice patients in the United States

**DOI:** 10.1017/ash.2025.10104

**Published:** 2025-09-05

**Authors:** Kimberlee Fong, Gurjit Brar, Wei Wei, Xiaoying Chen, Anu Shrestha, Renato Samala

**Affiliations:** 1 Department of Palliative and Supportive Care, The Lois U. and Harry R. Horvitz Palliative Medicine Program, Taussig Cancer Institute, Cleveland Clinic, Cleveland, OH, USA; 2 Department of Quantitative Health Sciences, Lerner Research Institute, Cleveland Clinic, Cleveland, OH, USA

## Abstract

**Background:**

Antimicrobials are frequently prescribed to hospice patients despite limited data on their utility.^1–3^ The Palliative Performance Scale (PPS) has been used for survival prediction among cancer patients and further generalized to end-of-life (EOL) diagnoses.^4^ This study aims to identify characteristics associated with antimicrobial usage within 30 days of EOL in non-hospitalized outpatient hospice patients from a single center in the United States (US).

**Methods/study design:**

We analyzed data on 1,111 hospice deaths from 2019. From these data, patients were divided into two groups: those who received antimicrobials at EOL (n = 212) and equally randomly computer-generated control group who did not. Fisher’s exact test and Wilcoxon rank sum test were used for analysis. PPS was recorded and used to determine functional status; higher PPS equates to higher functional status. Multivariable logistic regression correlated patient characteristics with EOL antimicrobial status.

**Results:**

Higher PPS scores were significantly associated with increased likelihood of antimicrobial use (Odds Ratio [OR] 1.40, 95% Confidence Interval [CI] 1.16–1.70). Male patients (OR 0.60, 95% CI 0.40–0.90) and patients with cancer (OR 0.61, 95% CI 0.39–0.96) were associated with lower odds of receiving antimicrobials. No significant association was found with age, race/ethnicity, residence, illness, or code status.

**Conclusion:**

The study identifies an association between PPS and antimicrobial prescribing near EOL. Tailoring antimicrobial use based on individual patient characteristics and goals may better align with hospice care objectives and aid in stewardship endeavors. Further research is needed to explore PPS as a potential tool to guide prescribing.

## Introduction

Hospice focuses on compassionate care at the end of a person’s life.^1^ The utilization and integration of hospice has grown tremendously in the past few years.^1,2^ In the United States (US), enrollment in hospice requires a diagnosis that results in an anticipated life expectancy of six months or less, and patients have chosen to forgo curative treatments in favor of comfort-focused measures.^3^ Similar to other medical care, hospice creates an environment where symptom management is prioritized and patients are not prescribed medications that do not provide benefit in their situation. One class of medications prescribed during hospice care is antimicrobials. However, currently there are no official guidelines to assist providers in facilitating prescribing decisions.^4,5^ It is important to define prescribing factors for the administration of antimicrobial medications as this will guide prescribing decisions. For the purpose of this study, antibiotics, antifungals, and antivirals, comprise the term antimicrobials.

Antimicrobial use is prevalent in end-of-life (EOL) hospice care. One of the first studies revealed that 27% of patients received antimicrobials during the last seven days of life yet only 15% of patients had an infectious indication.^6^ Previous research has also examined antimicrobial prescriptions during the last 7–14 days of life.^6–8^ Virik and Glare^9^ validated the PPS upon admission to an inpatient specialist palliative care unit finding a median postadmission survival to be 29.1 days which informed our focus on the last 30 days of life in this study. Despite utilization of antimicrobials, data is limited on the effectiveness, limitations, and indications for antimicrobial use among hospice patients.^10–12^ There are no clear guidelines on appropriate use of antimicrobials during EOL.^4,13^ It is crucial to remember that antimicrobials are not harmless; patients may develop adverse reactions such as *Clostridioides difficile* infection, allergic reaction, or create antimicrobial resistance.^14–17^

Physicians can utilize a variety of objective measures to assess their patients. Objective tools such as the Eastern Cooperative Oncology Group performance status (ECOG) and Karnofsky Performance Scale (KPS) are used by oncologists when deciding cancer therapy. Similarly, the Palliative Performance Scale (PPS) is a reliable tool that predicts survival among cancer patients and has been further generalized to other terminal diagnoses,^18,19^ wherein higher PPS scores are associated with higher functional status. The Association for Professionals in Infection Control and Epidemiology (APIC) and the Healthcare Infection Control Practices Advisory Committee (HICPAC) have published definitions and criteria for infections among home hospice patients. To our knowledge, there has not been a study reviewing antimicrobial use associated with PPS functional status in the US.

There is a growing need for antimicrobial stewardship in hospice as well as the field of palliative medicine.^20,21^ With ever-evolving antimicrobial resistance, identifying characteristics among non-hospitalized outpatient hospice patients who received antimicrobials may assist providers in deciding patient appropriateness for antimicrobials during EOL and maintain consistency. Therefore, this study is aimed at providing more insight into what factors contribute to antimicrobial prescribing in outpatient, non-hospitalized hospice care to further improve antimicrobial guidelines.

## Methods

### Study design and setting

We conducted a retrospective chart review of patients who died while receiving care from Cleveland Clinic Hospice (CCH) between January 1, 2019, and December 31, 2019. CCH provides inpatient and outpatient hospice services across the Cleveland Clinic Health System, with an average daily census of 250–300 patients. In this study, we reviewed hospice deaths where antimicrobials were prescribed in the last 30 days of life^9^ to non-hospitalized outpatient hospice patients. Data, such as demographics (gender, race/ethnicity, residence), PPS score, hospice diagnosis, illness, code status, and presence of advanced directives, such as living wills or power of attorneys, were obtained. These predictors were chosen based on prior studies reviewing antimicrobial use in EOL care.^22,23^ This retrospective chart review utilizing decedent data did not require Institutional Review Board (IRB) approval.

### Study population and data collection

We identified all patients who received antimicrobial prescriptions within their last 30 days of life and died in a non-hospital outpatient care setting. Patients who elected transitioning to inpatient hospice and subsequently died in the hospital were excluded to decrease confounding variables. Three US fellowship trained hospice and palliative medicine authors (KSF, AS, RVS) reviewed an equal number of patient charts to collect demographics, antimicrobial prescription details, prescriber information, hospice diagnosis, infection diagnoses, and PPS scores. Infection diagnoses were determined through hospice caregiver documentation of signs, symptoms, or specific infection diagnoses with International Classification of Diseases Tenth Revision (ICD-10) codes located in their medical records.

### Variables of interest

The variables of interest were: (1) characteristics of patients receiving antimicrobial prescriptions and (2) comparison of functional status, using PPS scores, between patients who did and did not receive antimicrobials. PPS scores range from 0% (death) to 100% (fully functional), with each 10% decrease representing a decline in physical function.^4,5^ We used the PPS score recorded closest to the antimicrobial written prescription date.

### Statistical analysis

A computer-generated random group of controls equal to the number of our case patients were selected from the remaining group of decedents who did not receive antimicrobial prescriptions. Categorical variables were summarized using frequencies and percentages, and continuous variables using median, IQR, and range. Fisher’s exact test and Wilcoxon rank-sum test were used for categorical and continuous variables, respectively. A multivariable logistic regression model assessed the association between prespecified patient characteristics and antimicrobial prescription status. A univariable analysis estimated the association between each predictor and outcome. A Research Electronic Data Capture (REDCap) platform was utilized to securely collect, store and manage data. All tests were two-sided with significance set at p≤.05. Analysis was performed using SAS Studio 3.7 (SAS Institute, Cary, NC).

## Results

Among 1,111 patients who died while receiving CCH care in 2019, 212 patients (19.1%) received antimicrobial prescriptions in their last 30 days of life while admitted to a non-hospitalized outpatient setting. This accounted for 272 total prescriptions as some patients received more than one prescription. Additionally, there were 212 random computer-generated group of controls that were selected from the remaining group of decedents who did not receive antimicrobial prescriptions.

Patient characteristics were evaluated as seen in Table 1. Antibacterials were most frequently prescribed (62%), followed by antifungals (36%), and antivirals (2%). Of the documented indications for antimicrobial use (n = 169, 62.1%), the most common were skin/wound symptoms (18.8%), genitourinary symptoms (14.3%), and respiratory symptoms (9.9%). Seven prescriptions were for continued prophylaxis in immunocompromised patients. In 103 cases (37.9%), the indication for antimicrobial prescription was not documented.

**Table 1.** Demonstrating patient characteristics in the study. *Unknown code status was not included in analysis. Hospice facility refers to a dedicated hospice residential center

	Antimicrobials prescribed in the last 30 days of life				All		Fisher’s exact test *P*-value
	No		Yes				
	N	%	N	%	N	%	
Gender							.03
Male	105	50	82	39	187	44	
Female	107	50	130	61	237	56	
Race/Ethnicity							.15
African American	41	19	39	18	80	19	
Caucasian	160	75	167	79	327	77	
Hispanic	2	1	4	2	6	1	
Other	9	4	2	1	11	3	
Primary hospice diagnosis							.17
Cancer	124	58	109	51	233	55	
Non-Cancer	88	42	103	49	191	45	
Non-Cance diagnosis							.99
GI disease	2	1	2	1	4	1	
Kidney disease	3	1	4	2	7	2	
Heart disease	29	14	29	14	58	14	
Liver disease	1	0	3	1	4	1	
Dementia	12	6	16	8	28	7	
ALS	1	0	2	1	3	1	
Non-Dementia Neurologic disorder	17	8	23	11	40	9	
Lung disease	17	8	19	9	36	8	
Other	6	3	5	2	11	3	
Code status							.49
Full code	5	2	8	4	13	3	
DNR-CCA	19	9	24	11	43	10	
DNR-CC	187	88	180	85	367	87	
Unknown*	1	0	0	0	1	0	
Presence of advanced directives							
Yes	212	100	212	100	424	100	
Place of residence							.69
Home	143	67	137	65	280	66	
Nursing facility	59	28	67	32	126	30	
Hospice facility	10	5	8	4	18	4	
Total	212		212		424		

Cancer was the predominant diagnosis (n = 109, 51.4%) among patients receiving antimicrobials, followed by heart disease (13.7%), non-dementia neurologic disease (10.9%), lung disease (9.0%), and dementia (7.5%). Cancer sub-types included: blood (n = 11), neurologic (n = 2), breast (n = 6), gastrointestinal (n = 35), genitourinary (n = 11), gynecologic (n = 8), lung (n = 23), skin (n = 2), and unknown/miscellaneous (n = 11), which included cancers of unknown origin. Non-hospice fellowship trained physicians wrote 60% of prescriptions, while hospice-trained or in-training physicians wrote 38% and non-physician advanced practice providers comprised 2% of prescribers.

Multivariable analysis revealed three significant associations with antimicrobial prescribing: Female gender (OR 1.67, 95% CI 1.11–2.52), and higher PPS score [PPS 40 vs 30] (OR 1.40, 95% CI 1.16–1.70), were associated with increased likelihood of antimicrobial prescriptions whereas cancer diagnosis (OR .61, 95% CI .39–.96) was associated with decreased likelihood of antimicrobials (Table 2). Other demographic factors, including age, race/ethnicity, patient residence, illness, and code status/resuscitation, showed no significant associations (Tables 2 and 3). A summary of the multivariable and univariable analysis is seen in Table 2 while Supplemental Figure 1 demonstrates the above characteristics in a Forest Plot for better clarity (supplemental data).

**Table 2.** Summary of multivariable and univariable logistic regression model for antimicrobial use status during the last 30 days of life. Hospice refers to a dedicated hospice residential center

		Multivariable logistic (n = 410)		Univariable logistic (n = 410)	
Factor	Comparison	Odds Ratio	95% CI	Odds Ratio	95% CI
PPS	10 points increase	1.40	1.16–1.70	1.30	1.10–1.56
Age	1 year increase	1.00	0.98–1.01	1.00	0.99–1.02
Gender	Male vs Female	.60	0.40–0.90	0.64	0.44–.94
Race/Ethnicity	Black vs White	.49	0.06–2.85	0.91	0.56–1.49
	Hispanic vs White	.49	0.06–2.74	1.92	0.37–13.95
Cancer	Yes vs No	.61	0.39–0.96	0.75	0.51–1.10
Residence	Home vs Hospice	1.63	0.59–4.74	1.20	0.46–3.22
	Nursing Facility vs Hospice	1.89	0.65–5.75	1.42	0.53–3.94
Code	Full Code vs DNR-CC	2.05	0.60–8.14	1.66	0.54–5.59
	DNR-CCA vs DNR-CC	1.18	0.61–2.33	1.31	0.68–2.50

**Table 3.** Evaluating age, PPS, and length of stay between antimicrobial group at EOL compared to those that did not receive antimicrobials at EOL, *P* value via Wilcoxon rank test

	Antibiotics prescribed in the last 30 days of life	N	Min	25^th^ percentile	Median	75^th^ percentile	Max	Wilcoxon rank Test P-value
Age at death (years)	No	212	27	66.05	78.15	88.15	100.9	.99
	Yes	212	32.9	66.2	77.35	89.2	103.6	
PPS score at 30 days prior to death	No	210	10	30	30	40	70	.001
	Yes	212	10	30	40	40	60	
Length of stay in hospice (days)	No	212	1	7	18	51.5	607	.68
	Yes	212	2	9	17	29	1 283	

## Discussion

Our study examined antimicrobial prescribing patterns near EOL in non-hospitalized outpatient hospice care patients. We found that 19.1% of patients received antimicrobials in their final 30 days of life. Previous studies performed in various clinical settings have documented antimicrobial use at EOL ranging from 17.6 to 63%.^6–8,11,24,25^ Baghban and Juthani-Meta^26^ proposed an algorithm that incorporates goals of care discussions to guide antimicrobial use at EOL. By utilizing the PPS to assess prognosis and subsequently functional status decline, providers may identify those patients approaching EOL. This study offers observations on the association between a person’s functional status and antimicrobial prescribing in EOL care. Implementing PPS scores and goals of care conversations can inform antimicrobial prescribing patterns and stewardship programs.

Patients with higher functional status, as measured by PPS, were associated with an increased likelihood of receiving antimicrobial prescriptions (OR 1.40, 95% CI 1.16–1.70). The functional difference between a PPS of 30 versus 40 is clinically significant; patients with PPS 30 are completely bedbound, require total care in their activities of daily living, and have reduced oral intake.^18^ The association observed in our study suggests a pattern where functional status may influence providers’ prescribing decisions. This observation aligns with findings by Pereira et al.^27^ where infections were not treated due to underlying poor general condition and imminent death as well as Crowley et al.^7^ where hospice patients with decreased survival time are less likely to receive antimicrobials. In hospice care, quality of life is the primary goal, therefore medications should be reviewed for their usefulness prior to prescribing.

In our study, cancer patients were associated with a lower likelihood of receiving antimicrobial prescriptions when compared to non-cancer diagnoses (OR .61, 95% CI .39–.96). This aligns with findings from White et al.^28^ where patients with advanced cancers often chose limited use of antimicrobials when goals of care conversations occurred with their care providers.

The gender disparity with women more likely to receive antimicrobials (OR 1.67, 95% CI, 1.11–2.52) mirrors community prescribing patterns described by Schroder et al.^29^ This may be partially explained by the observed difference in urinary tract infection (UTI) prevalence between females and males. According to Vittetta et al.^30^ UTIs were the most treated source of infections in hospice patients. Documentation of urinary symptoms in females occurred more frequently in our cohort. Additionally, Lau et al.^19^ surmised that females had increased survival time therefore higher functional status as was measured by the PPS.

### Antimicrobial stewardship implications

Documentation of specific infectious indications was present in 62.1% of prescriptions (n = 169), with skin/wound (n = 51, 18.8%), genitourinary (n = 39, 14.3%) and respiratory symptoms (n = 27, 9.9%) being the most common. These align with Clark et al.^23^ where the three main symptoms being treated were similar to our study. There were 7 antimicrobial prescriptions that were continued as prophylaxis for an underlying immunocompromised state and raises further questions about appropriate discontinuation of antimicrobials near EOL. There was a lack of documented antimicrobial indications for 37.9% of prescriptions which highlights a gap in antimicrobial stewardship practices. This finding is particularly concerning as many studies have discussed over prescription of medications^31,32^ and most prescriptions in our study (60%) were written by non-hospice fellowship trained providers.

The rationale for decreasing antimicrobial overuse near EOL extends beyond individual patient care. Unnecessary exposure to antimicrobials can lead to adverse effects such as allergic reaction, antimicrobial associated diarrhea, *Clostridioides difficile*, and unnecessary side effects in an already debilitated population.^14–17^ Moreover, varied antimicrobial use may lead to development of multidrug-resistant organisms posing a significant threat to public health.^33^ Of note was the number of prescriptions for skin/wound symptoms utilizing antibiotics and antifungals. The Centers for Disease Control and Prevention (CDC) has urged physicians to be judicious in prescribing antifungals in light of the emergence of resistant skin infections.^34^

### Study limitations

Our retrospective design limited our ability to assess symptomatic improvement following antimicrobial therapy. Inconsistent documentation also challenged our ability to determine an infectious diagnosis, which was also noted in previous studies by D’gata et al.^35^ Clark et al.^23^ and Albrecht et al.^6^ Additionally, as we reviewed prescription data, this may not reflect actual patient medication usage or adherence. Another limitation was the study was conducted in a single hospice system thus narrowing our study population. Furthermore, the lack of objective assessment of appropriateness could be considered a limitation as our data did not determine if antimicrobials were truly indicated.

### Future directions

This study is the first to review antimicrobial prescribing in relation to functional status using PPS scores in non-hospitalized, outpatient hospice care. The PPS is validated for assisting in prognostication of diseases in hospice^18^ and is currently utilized by American hospice interdisciplinary teams. Antimicrobial stewardship guidelines already exist for hospital systems and long-term care facilities.^36^ Our findings suggest a multilevel approach to improve antimicrobial stewardship including: development of PPS-based antimicrobial prescribing guidelines, implementation of standardized documentation requirements for prescriptions, creation of education programs (especially focusing on non-hospice fellowship trained providers), and establishing criteria for antimicrobial discontinuation in EOL care. Stewardship interventions utilizing PPS in the non-hospitalized outpatient hospice setting could enhance appropriate use of antimicrobials near EOL.

## Conclusion

This case-control study identified associations between patient characteristics and antimicrobial prescribing patterns near EOL in non-hospitalized outpatient hospice care. Patients with higher functional status as measured by PPS tend to be more likely to receive antimicrobial prescriptions. Overall, PPS is associated with antimicrobial prescribing and could be explored in future prospective studies as a potential tool to help guide prescribing practices.

## Supporting information

10.1017/ash.2025.10104.sm001 Fong et al. supplementary material Fong et al. supplementary material

## Supplementary material

To view supplementary material for this article, please visit https://doi.org/10.1017/ash.2025.10104.
