# Supplementary figures and images for: A case-control study of end-of-life antimicrobial use in Non-hospitalized hospice patients in the United States
Source: Antimicrob Steward Healthc Epidemiol. 2025 Sep 5;5(1):e201. doi: 10.1017/ash.2025.10104 (PMC12415798; doi:10.1017/ash.2025.10104)

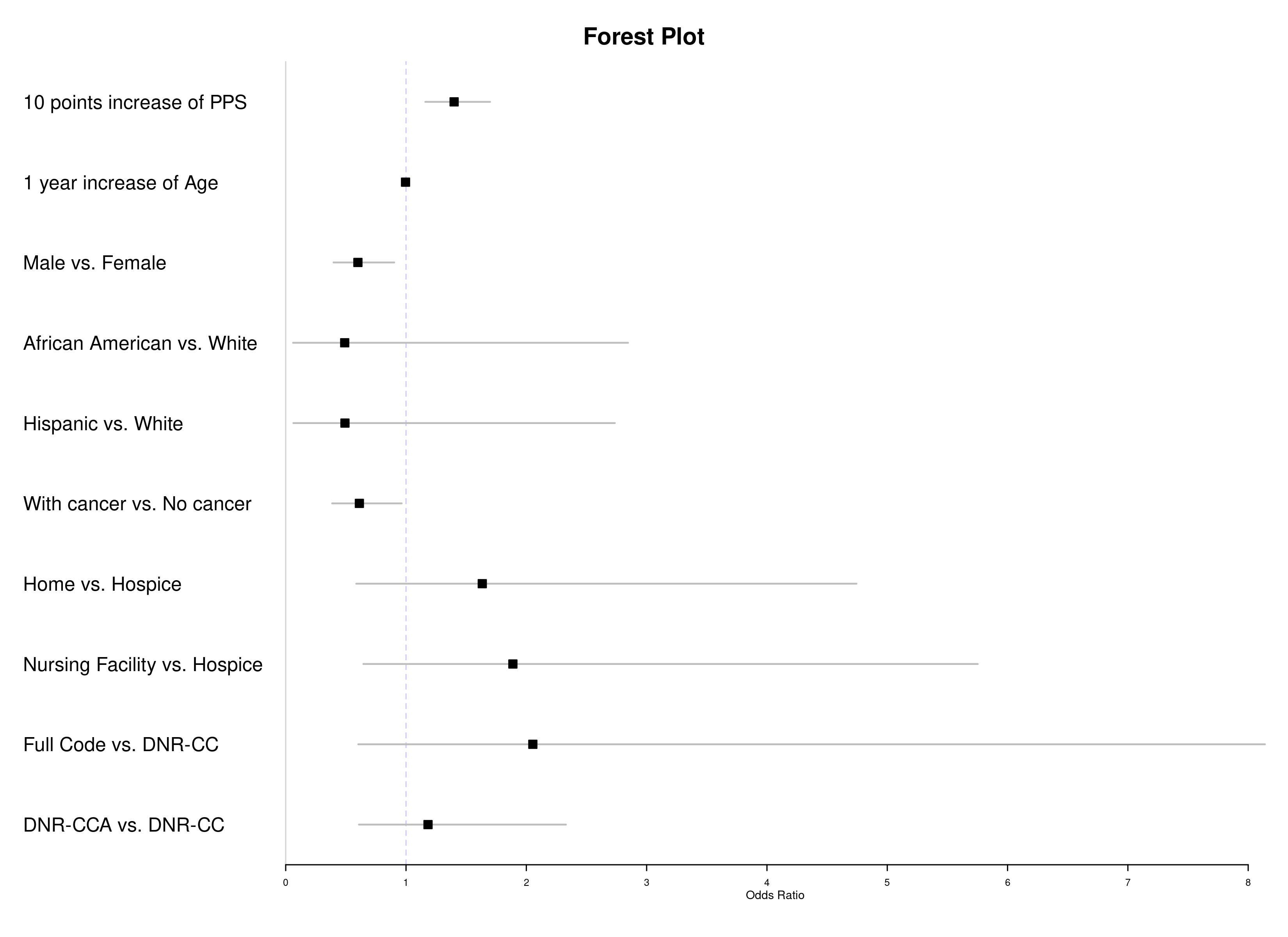

Supplement: Fong et al. supplementary material [file S2732494X25101046sup001.tiff]
